# Smartphone-Supported Vestibular Rehabilitation in Individuals With Vestibular Dysfunction: Pilot Randomized Crossover Trial Assessing Functional Clinical Outcomes and Anxiety

**DOI:** 10.2196/84207

**Published:** 2026-03-24

**Authors:** Azriel Kaplan, Liran Kalderon, Amit Wolfovitz, Yoav Gimmon, Shelly Levy-Tzedek

**Affiliations:** 1 Department of Physical Therapy, Recanati School for Community Health Professions, Ben-Gurion University of the Negev, Beersheva, Israel; 2 Department of Otolaryngology—Head and Neck Surgery, Sheba Tel-HaShomer Medical Centre, Ramat Gan, Israel; 3 Department of Physical Therapy, Faculty of Social Welfare & Health Sciences, University of Haifa, Eshkol Tower, Department of Physical Therapy, Haifa, 3103301, Israel, 972 506442243; 4 Zelman Center for Neuroscience, Ben-Gurion University of the Negev, Beersheva, Israel; 5 Freiburg Institute for Advanced Studies (FRIAS), University of Freiburg, Freiburg, Germany

**Keywords:** vestibular rehabilitation, dizziness, vestibular application, vestibular anxiety, vestibular technology, rehabilitation technology, mobile phone

## Abstract

**Background:**

Vestibular disorders impair balance, increase fall risk, and reduce quality of life due to dizziness and vertigo. They are frequently accompanied by heightened anxiety, which may further limit daily functioning and contribute to avoidance behaviors. Although vestibular rehabilitation has been extensively studied and shown to be effective in managing vestibular disorders, adherence to home-based exercises remains low for many dizzy patients. This is often attributed to uncertainty about correct performance, lack of feedback, or difficulty maintaining a structured routine. To help address these barriers, Vestibulon, a smartphone app co-designed with clinicians and patients, was developed to support rehabilitation practice by providing guided exercise, structured scheduling, progress monitoring, and clear instructions intended to promote confidence and engagement.

**Objective:**

We aim to evaluate the potential contribution of a smartphone-based app to vestibular rehabilitation outcomes and to explore the relationship between dizziness-related disability and anxiety during the intervention.

**Methods:**

This randomized 2-period crossover pilot trial included 20 adults with vestibular dysfunction (mean age 52, SD 12 y) who completed 6 weeks of rehabilitation. Participants were randomized to begin with either app-supported or conventional treatment before crossing over to the alternate condition after 3 weeks. This design enabled each participant to experience both modes of rehabilitation. Assessments were conducted at baseline (T0), mid-study (after 3 wk; T1), and end of study (T2). Outcome measures included the Dizziness Handicap Inventory (DHI) to assess dizziness-related disability, the State-Trait Anxiety Inventory to evaluate anxiety, and the Instrumented Timed Up and Go test to quantify functional mobility. The primary outcome was the change in DHI scores across time points.

**Results:**

Significant improvements in DHI were observed between T0 and T2 in both groups (median change: app first=34, IQR 6‐35 points, *P*=.006; conventional first=18, IQR 10‐19 points, *P*=.009). Improvement in Instrumented Timed Up and Go performance was observed only when the app-supported phase occurred first (Z=−2.45, *P*=.01), suggesting a potential early benefit of structured smartphone guidance. State-Trait Anxiety Inventory scores did not change significantly in either sequence. Across all time points, dizziness-related disability and state anxiety demonstrated a consistent and significant moderate correlation (*r*=0.64, *P*<.001), emphasizing the strong interplay between physical and psychological symptoms in individuals with vestibular disorders.

**Conclusions:**

This pilot study indicates that smartphone-supported vestibular rehabilitation has the potential to enhance functional outcomes for some patients. The consistent association between dizziness and anxiety underscores the relevance of considering psychological factors in vestibular rehabilitation. Given the preliminary nature of this study and the small sample size, these findings should be interpreted cautiously, and further research is needed to determine the app’s effectiveness in larger randomized controlled trials.

## Introduction

Central or peripheral vestibular dysfunction may cause dizziness, vertigo, and unsteadiness [1]. The peripheral injury can be classified as either unilateral or bilateral vestibular hypofunction [2]. In 2014, a total of 53‐95 million people across the United States and Europe endured vestibular hypofunction [3]. The common symptoms are vertigo, dizziness, instability, ataxia, nystagmus, and impaired navigational abilities [1,4,5]. A decrease in balance caused by vestibular hypofunction significantly increases the risk of falling in active older adults [6]. The symptoms of vestibular hypofunction may cause impairment in participation in social gatherings and events, quality of life, and a feeling of anxiety [7,8].

Alongside its physical and functional effects, dizziness has been associated with psychological factors, particularly anxiety [7,9,10], although the mechanisms underlying this association are still being investigated. Recent evidence suggests that cerebellar involvement in vestibular processing may play a role in this relationship [11]. Accordingly, interactions between the vestibular system and the cerebellum could be key to understanding the relationship between balance issues and anxiety disorders [11]. In addition, several studies have shown that the scores of the Dizziness Handicap Inventory (DHI) questionnaire are correlated with various anxiety questionnaires, but most of these studies examined specific vestibular populations, such as people with vestibular migraine, or measured the correlation at only one point in time [12-14].

Over the years, several studies [15-18] have shown the safety and effectiveness of vestibular rehabilitation. In vestibular rehabilitation, exercises are performed to alleviate symptoms with habituation exercises, improve gaze stability with adaptation and substitution exercises, and improve balance [15]. According to the clinical practice guidelines for vestibular rehabilitation published by the American Physical Therapy Association and updated in 2022, it is recommended to practice 3 to 5 times per day for a total of 20‐40 minutes daily over 4 to 6 weeks [15].

Despite vestibular rehabilitation’s effectiveness and safety, patients report difficulty during the process, including time commitment and difficulty getting to many face-to-face meetings with the therapist [19]. Moreover, access to vestibular rehabilitation is limited (eg, in terms of distance from the clinic or clinician availability); in a European survey among vestibular therapists, 48% described accessibility as difficult or very difficult [20].

In recent years, studies have examined several ways to make vestibular practice more accessible, such as through practicing tai chi or using virtual reality in vestibular rehabilitation [21]. These methods have shown efficacy in some studies [22,23]. Despite this, these methods are rarely used in practice, possibly because it can be challenging to combine short periods of exercise several times a day [24], as recommended in clinical practice guidelines. In addition, the high cost of robotic [25] or virtual-reality [22] systems may also account for the absence of these technologies from the rehabilitation landscape [22].

Several phone applications have been developed in recent years to aid in vestibular rehabilitation [26-28]. DSilva et al [26] and Nehrujee et al [27] examined the usability of the application after a 1-time experience on individuals with vestibular disorders as well as on healthy individuals. Hovareshti et al [28] validated the performance of a tablet application (which can also be adapted for mobile phones) designed for monitoring practice while identifying head movements and gaze focus using the camera. In addition, they also presented a case series examining the effect on eye-gaze accuracy of 3 patients and 1 healthy individual who used the application [29]. However, these studies did not examine the effects of the phone applications’ impact on patients’ clinical outcomes over the course of the vestibular rehabilitation program.

To meet the emerging need and address the existing difficulties in the accessibility of vestibular rehabilitation [19,20,24], we developed the “Vestibulon” phone app, aimed to help patients perform their vestibular exercises; the app provides feedback for the user, gives a clear explanation of each exercise, helps manage practice time, and provides information and feedback to the therapist and patient.

The first goal of this research is to examine the effect of practice with the “Vestibulon” app on functional balance and anxiety indices. These were measured using the following parameters: (1) Instrumented Timed Up and Go Test (iTUG), (2) DHI, and (3) State-Trait Anxiety Inventory (STAI). The second goal is to assess the association between balance function (iTUG) and anxiety (STAI). We hypothesized that practice supported by the app would increase compliance with the practice, thereby improving indicators of balance and anxiety.

## Methods

### Study Design

This study was conducted using a randomized crossover design. Each participant was randomly assigned to 1 of 2 groups, while gender variables were stratified to ensure an equal distribution. A computer program determined the randomization into groups. Due to the nature of the intervention, outcome assessors were not blinded; no blinding of participants or therapists was possible, and allocation was not concealed. A total of 6 weeks of vestibular rehabilitation were given to each participant. During the first period, which lasted 3 weeks, group 1 used the app for their vestibular rehabilitation, and group 2 did their vestibular rehabilitation exercises without the app. At the crossover point, they switched, such that in the second period, which also lasted 3 weeks, group 1 did their rehabilitation exercises without the app, and group 2 used the app. Measurement sessions were held at Sheba Hospital’s otolaryngology clinic between June 2023 and July 2024. This study was concluded in July 2024, following completion of planned recruitment. All study participants gave their written informed consent. This trial is reported in accordance with the CONSORT (Consolidated Standards of Reporting Trials) 2010 statement: extension to randomized crossover trials (Checklist 1).

### Participants

A total of 26 participants were randomly assigned to the study. Further, 6 participants dropped out during the study, resulting in 20 participants completing this pilot study. As this is a preliminary investigation, a formal power analysis was not performed. Instead, our sample size aligns with literature suggesting a minimum of 12 participants per arm to adequately gauge feasibility and variance for future calculations [30]. In this crossover design, participants received both interventions, ensuring that the final sample exceeded this minimum requirement per arm. A broad age range (18‐75 y) was intentionally included to allow an initial feasibility assessment across diverse adult users, in line with the aims of a pilot study.

### Inclusion and Exclusion Criteria

The inclusion criteria were (1) participants’ age of 18‐75 years, (2) diagnosis of vestibular dysfunction, and (3) being fluent in Hebrew. The exclusion criteria were (1) a medical condition that prevents the participant from performing home vestibular rehabilitation practice, such as orthopedic, neurological, cardiac, or visual impairment; (2) individuals with dizziness who have already been given a home vestibular rehabilitation exercise program; and (3) diagnosis of central vestibular disorder due to brain structural damage.

### Recruitment

Participants were recruited through the Sheba Hospital Dizziness Clinic. Clinicians informed eligible patients about this study during routine visits and provided them with a study information sheet. Those interested provided their contact details, and a research team member subsequently contacted them to explain this study further, confirm eligibility, and schedule the baseline assessment.

### Ethical Considerations

All study procedures were approved by the Helsinki Committee of Sheba Medical Center (protocol number 9576‐22-SMC), and the trial was prospectively registered on ClinicalTrials.gov (NCT05959278; registration date: 07/06/2023). All participants provided written informed consent before enrollment and were informed of their right to withdraw from this study at any time without consequence. No secondary use of data from prior studies was involved, and thus no additional waivers or exemptions were required.

To ensure privacy and confidentiality, all data collected for this study were anonymized using coded identifiers. No personally identifying information was stored within the research database or within the investigational app. Paper-based documents (eg, consent forms) were stored in a locked cabinet inside a locked research laboratory, and all digital data were stored on secure, access-restricted institutional servers. Only authorized study personnel had access to coded datasets. Participants did not receive financial compensation for participation in this study.

### Outcome Measures and Data Collection

The investigational smartphone app, Vestibulon, was designed to support the delivery of conventional vestibular rehabilitation remotely, with an emphasis on accessibility, clarity, and adherence. Its development was informed by a participatory design process, incorporating insights from patients and clinicians regarding usability, clarity of instructions, accessibility, and barriers to home-based vestibular rehabilitation [25]. These stakeholder inputs directly shaped core features of the app, including its simple interface, structured reminders, and emphasis on supporting daily adherence to standard vestibular exercises. All participants used the same version of the app during this study.

Vestibulon enables the clinician to predefine the rehabilitation parameters for each user, including the type of exercise (adaptation, habituation, and substitution), frequency, intensity, and recommended duration, based on the patient’s diagnosis and clinical characteristics. The exercises included in the app correspond to standard, widely accepted components of vestibular rehabilitation. Before each exercise, the app provides step-by-step written and visual instructions to ensure correct performance.

To promote adherence, the app delivers push-notification reminders for exercise sessions. Each user selects the preferred times of day for receiving these reminders and can modify these settings at any time during the intervention period. When a reminder appears, users can choose to begin the exercise, postpone it (“snooze”), or dismiss it. After completing an exercise session, the user is prompted to provide brief feedback (eg, whether the exercise was completed and whether they wish to continue). These features are intended to help patients structure their daily practice and maintain consistency across sessions. Participants received a brief standardized onboarding session of approximately 10 minutes, during which the app was installed and demonstrated, and users were instructed on selecting reminder times, navigating the exercise interface, and submitting feedback. No advanced technical skills were required to operate the app. The app functioned reliably throughout this trial, and no major technical issues or crashes were reported.

### Outcome Measures

#### Instrumented Timed Up and Go Test

The iTUG examines functional balance [31]. The test measures the time it takes for a participant to stand up from a seated position on a chair, walk for 3 meters at their own pace, turn around, return for 3 meters, turn again, and return to their seated position [31]. We used the iTUG for a quantitative assessment for this test [32]. For the measurement, we used Opal (APDM) sensors and the instructions in the APDM “mobility lab” application.

#### State-Trait Anxiety Inventory

The STAI is a self-report questionnaire designed to examine a person’s general and current anxiety levels [33]. The questionnaire is divided into 2 parts, each consisting of 20 statements, which the participant should rate on a scale of 1‐4 according to their degree of agreement [34]. This questionnaire has been used among patients who endure vestibular disorders [35]. We used this questionnaire to better distinguish between the participant’s general and momentary anxiety to assess the effect of the vestibular rehabilitation on anxiety levels.

#### Dizziness Handicap Inventory

The DHI is a 25-item questionnaire designed to assess the impact of dizziness on everyday life in people with vestibular disorders [36]. The questionnaire has 3 parts: physical, emotional, and functional [36]. The minimal clinically important difference (MCID) of the DHI is an 18-point difference [36]. Participants with a baseline DHI score of ≤17 were excluded from the MCID analysis because, given the 18-point MCID threshold, these individuals could not mathematically achieve an MCID improvement. Including them would introduce a floor effect and underestimate the proportion achieving clinically meaningful change. In 2010, a Hebrew translation of the questionnaire was published [37], and this is the version we administered to participants. No changes to outcome measures were made after trial commencement.

### Procedures

This was a 2-period, 2-sequence randomized crossover trial. A washout period was not implemented to avoid interrupting rehabilitation and potentially compromising clinical progress, although this may allow residual effects to influence the second phase. Each participant was administered home exercises for gaze stabilization and habituation for a total of 20‐30 minutes each day, across 7 to 10 sessions (2‐4 min per session). The exercises were administered by a vestibular physical therapist. Upon entry to this study (T0), we took the baseline measurements of the iTUG, DHI, and STAI. Participants were then randomized into 1 of 2 groups: one that practiced with the app first (AF), or one that started with conventional practice first (without an app; conventional first, CF). At the end of the first intervention period (T1), we performed the second set of measurements of the same parameters. This was the crossover point at which participants from each group switched to the alternative treatment condition. That is, during the second intervention period, the AF group received conventional treatment, and the CF group used the app to perform their rehabilitation exercises. Each intervention period lasted approximately 3 weeks, for a total of 6 weeks of rehabilitation per participant.

After the completion of both intervention periods (T2), we performed the endpoint measurement of the iTUG, DHI, and STAI. The 6-week period was chosen based on the clinical practice guidelines for vestibular rehabilitation [15]. Participants were aware of the intervention type. The app-based and conventional rehabilitation provided the same therapeutic content but differed in their mode of delivery.

### Data Analysis

We used the Kolmogorov-Smirnov test to examine the normal distribution of quantitative variables on the ratio scale.

For evaluating differences in the STAI, DHI, and iTUG indices between groups, we used the Mann-Whitney *U* test. To assess changes in STAI, DHI, and iTUG across the 3 time points (T0, T1, and T2), we applied the Friedman test. When significant differences were identified, post hoc analyses were conducted using the Wilcoxon signed-rank test, with Bonferroni correction applied to account for multiple comparisons. Finally, to explore potential correlations between the iTUG indices, DHI scores, and the STAI questionnaire, we calculated Spearman correlation coefficient. Statistical analysis was performed with SPSS (version 28; IBM Corp) software, and results were considered significant when *P* values were <.05.

## Results

### Participant Characteristics

**Figure 1.**
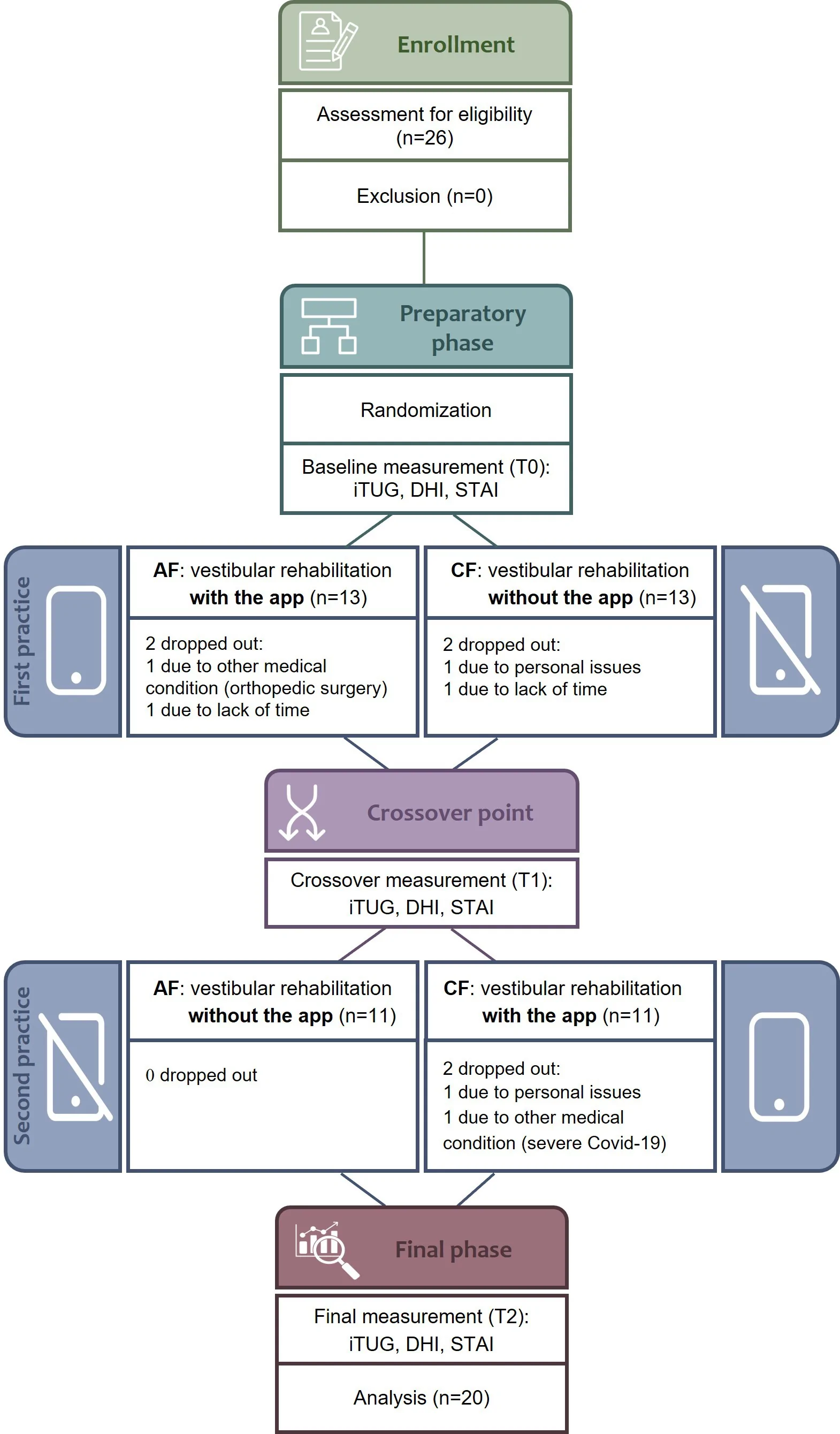
Study overview. The flowchart illustrates the participant enrollment, intervention groups (one that started with app assistance [AF] and one that started with conventional rehabilitation [CF]), and assessment time points. The app icon indicates the app-assisted rehabilitation period. AF: app first; CF: conventional first; DHI: Dizziness Handicap Inventory; iTUG: Instrumented Timed Up and Go; STAI: State Trait Anxiety Inventory.

A total of 26 participants were recruited for this study. Further, 20 participants (mean age 52.4, SD 12.6 y; 8 men and 12 women) completed this study (Figure 1, Table 1). There was no statistically significant difference in the baseline data between the 2 groups. Participants who completed this study presented with the following vestibular disorders: bilateral vestibular hypofunction (n=6), unilateral vestibular hypofunction (n=4), Meniere disease (n=3), vestibular migraine (n=2), persistent postural perceptual dizziness (PPPD; n=3), labyrinthitis (n=1), and postconcussive syndrome (n=1). Furthermore, 6 participants dropped out during this study (mean age 46.7, SD 14.0 y; 2 men and 4 women).

**Table 1.** Demographics and baseline questionnaire scores of each group. Baseline data of each group and the baseline data of all groups together. No statistically significant difference was found between the groups in any of the characteristics.

Characteristic	AF^a^ (n=11)	CF^b^ (n=9)	Total (N=20)	*P* value
Age (years), mean (SD)	48.5 (12.8)	57.3 (11.1)	52.4 (12.6)	.16^c^
Gender (woman/man), n (%)	7 (63.6) / 4 (36.4)	5 (55.6) / 4 (44.4)	12 (60) / 8 (40)	.53^d^
DHI^e^ total score, median (IQR)	54.0 (36-64)	44.0 (32-74)	49.0 (33-68)	.88^c^
DHI physical, median (IQR)	14.0 (9-19)	14.0 (12-18)	14.0 (9-18)	.91^c^
DHI functional, median (IQR)	18.0 (8-28)	20.0 (10-30)	19.0 (9-30)	.73^c^
DHI emotional, median (IQR)	20.0 (11-29)	14.0 (10-30)	17.0 (10-30)	.73^c^
STAI^f^ total, median (IQR)	83.0 (64-92)	92.0 (78-93)	87.0 (64-94)	.62^c^
STAI state, median (IQR)	42.0 (32-49)	44.0 (33-51)	43.0 (32-50)	.94^c^
STAI trait, median (IQR)	34.0 (30-50)	45.0 (34-55)	36.5 (31-54)	.67^c^
iTUG^g^ total time, mean (SD)	11.4 (2.2)	11.1 (1.5)	11.1 (1.8)	.79^c^

a AF: app first.

b CF: conventional first.

c Mann-Whitney test.

d Chi-square test (Fisher Exact Test).

e DHI: Dizziness Handicap Inventory.

f STAI: State Trait Anxiety Inventory.

g iTUG: Instrumented Timed Up and Go.

### Effects of the Phone App on Disability, Anxiety, and Functional Balance Measures

#### DHI Total

Both groups (AF and CF) show statistically significant differences between T0-T2 in the DHI total (AF: Z=−2.80, *P*=.006; CF: Z=−2.67, *P*=.009).

**Figure 2.**
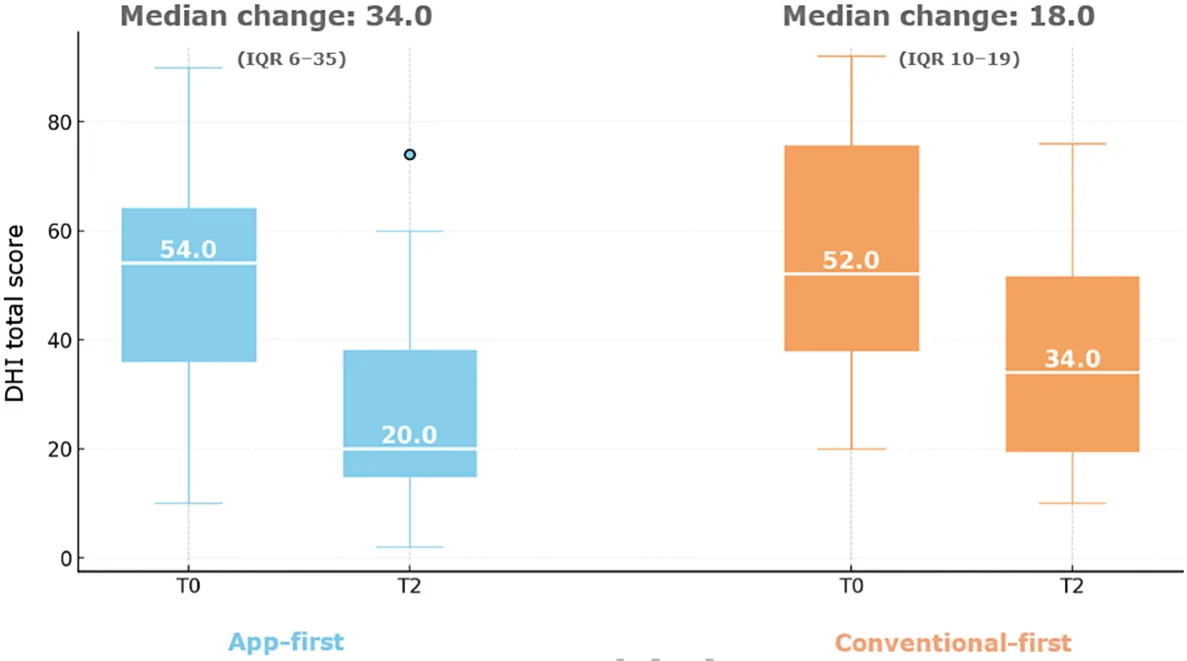
The change in median DHI total between T0-T2. Within each group (AF and CF), we found a statistically significant difference between T0 and T2. A blue dot indicates an outlier. Blue color represents the app-first group, and orange color represents the conventional-first group. The values in the box plot represent the median values at T0 (first evaluation when beginning the rehabilitation period) and at T2 (last evaluation after 6 weeks of rehabilitation). AF: app first; CF: conventional first; DHI: Dizziness Handicap Inventory.

The total DHI score decreased by 62.9% in the AF group and by 34.6% in the CF group between baseline and end-of-study; however, the difference between the groups was not statistically significant (Z=−0.80, *P*=.42). Figure 2 summarizes the change in DHI total.

**Figure 3.**
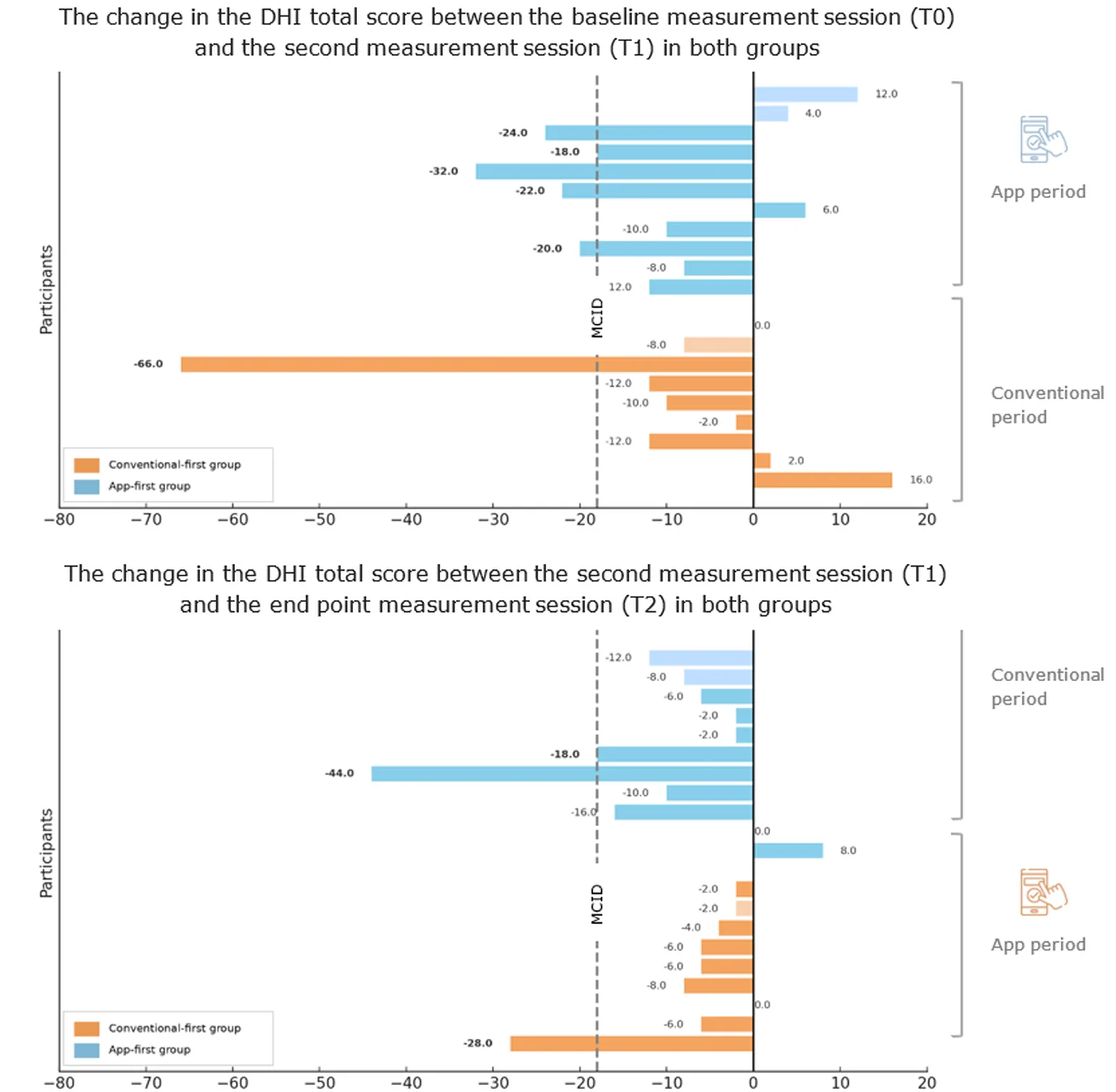
Change in DHI total score and number of patients achieving the minimal clinically important difference score. Top: the change in the DHI total score between the baseline measurement (first evaluation before the rehabilitation period [T0]) and the second measurement session (second evaluation after 3 weeks of rehabilitation [T1]) in both groups. Bottom: the change in the DHI total score between the second measurement session (T1) and the end point measurement session (last evaluation after 6 weeks of rehabilitation [T2]) in both groups. The results of the app-first group are marked in light blue; those of the conventional-first group are marked in orange; a phone-app icon indicates that the app was used during this period. The bolded numbers indicate that these participants’ score changes met or exceeded the MCID threshold. The lighter bars indicate participants with a floor effect, that is, whose initial DHI score was 17 or below. DHI: Dizziness Handicap Inventory; MCID: minimal clinically important difference.

Figure 3 illustrates the changes in the DHI total score during both practice periods, as well as the number of participants who reached or exceeded the MCID.

By the end of this study, 7 of 9 (77%) participants in the AF group exceeded the MCID threshold, compared to 2 of 8 (25%) participants in the CF group. This difference between the groups did not quite reach statistical significance (*χ*²_1_=4.97, *P*=.06).

When comparing the with-app practice period to the no-app practice period, regardless of the group (AF/CF), we found that 6 of 17 (35.2%) participants met or superseded the MCID threshold during the period with the app (T0-T1 for the AF group and T1-T2 for the CF group), while 3 of 17 (17.6%) participants met or superseded the MCID threshold without the app (T1-T2 for the AF group and T0-T1 for the CF group).

No statistically significant change was found between the 3 measurement sessions in the STAI state (*χ*^2^_2_=0.80, *P*=.67) or in the STAI trait (*χ*^2^_2_=1.71, *P*=.42) in the AF+CF together, and in each group separately.

**Figure 4.**
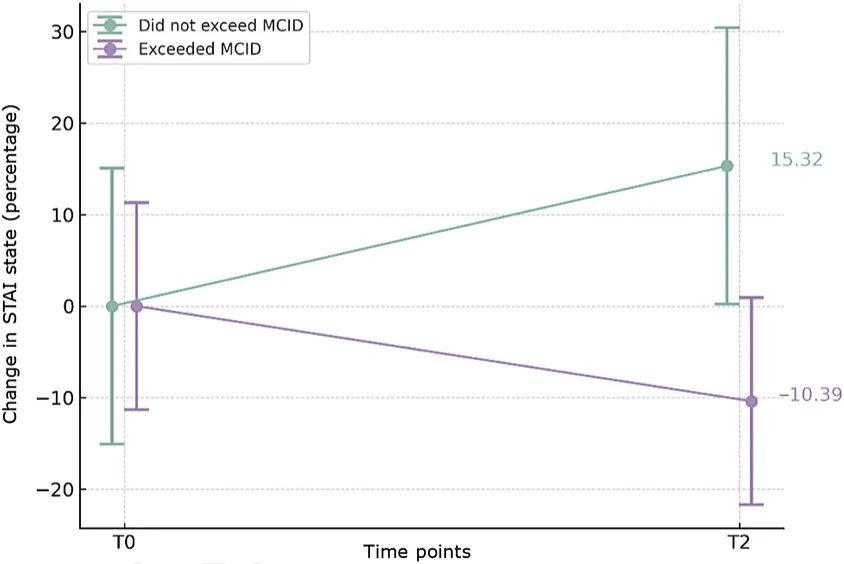
Contrasting trends in STAI State anxiety changes relative to DHI improvement. Participants who achieved or exceeded the MCID in DHI scores between T0 and T2 showed a trend towards lower STAI State scores. Conversely, those who did not reach the MCID in DHI improvement demonstrated a trend towards higher STAI State scores. DHI: Dizziness Handicap Inventory; MCID: minimal clinically important difference; STAI: State Trait Anxiety Inventory.

When we divided the data of all the participants into 2 groups: those who met or superseded the MCID threshold in the DHI total (a change of 18 points or more) and those who did not (a change of fewer than 18 points), we found opposing trends: the group that superseded the MCID showed a 10.3% decrease in the STAI state score between T0 and T2, while the group that did not pass the MCID showed an increase of 15.3% (Figure 4). This difference is not statistically significant (Z=−1.73, *P*=.09).

**Figure 5.**
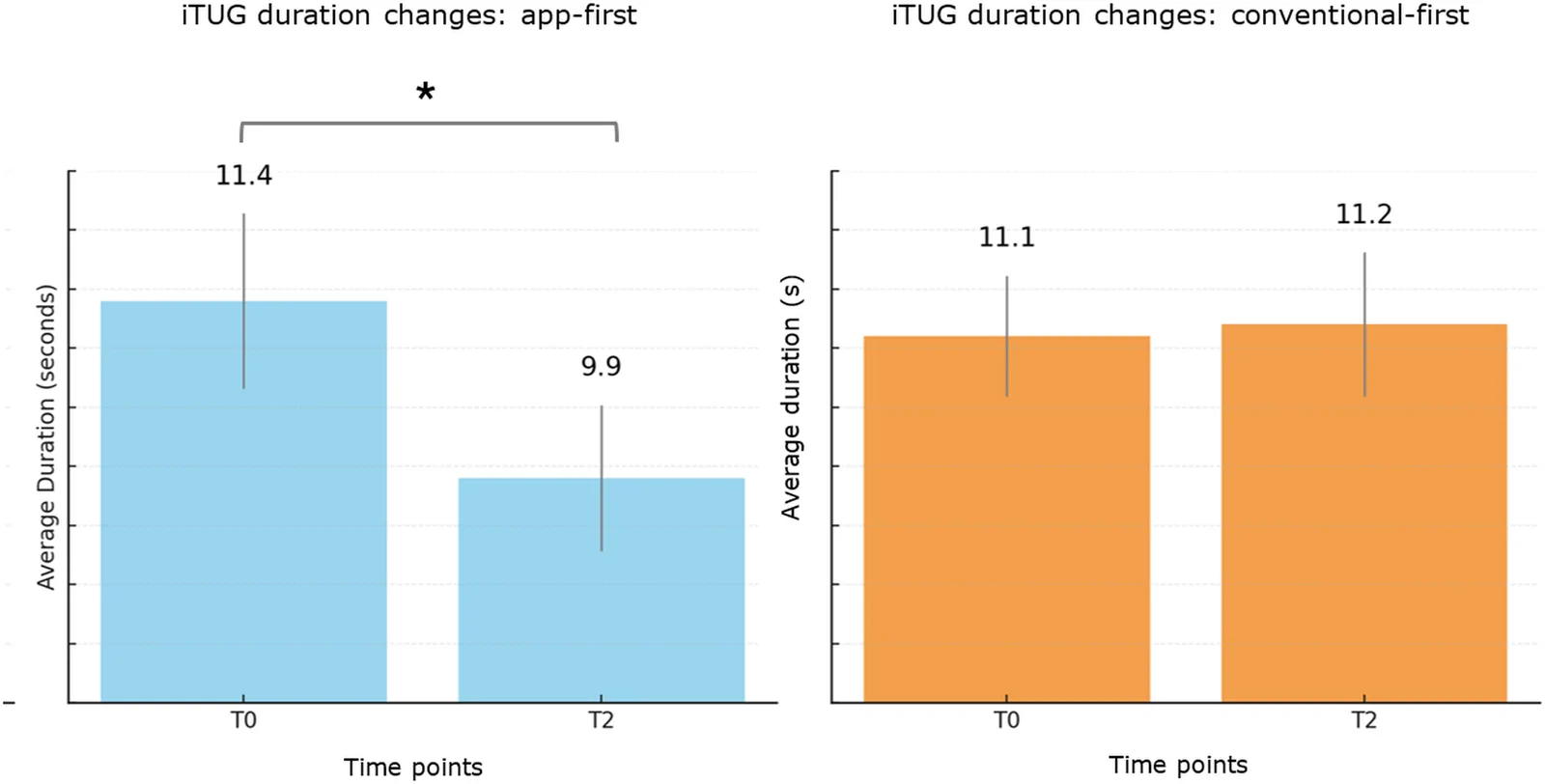
Change in iTUG total duration between baseline (first evaluation at the beginning of the rehabilitation period [T0]) and the end of this study (last evaluation after 6 weeks of rehabilitation [T2]) * indicates a statistically significant difference between the time points. iTUG: Instrumented Timed Up and Go.

In the AF group, a statistically significant change was found across the 3 iTUG measurements (*χ*^2^_2_=9.45, *P*=.009; Figure 5). We found a statistically significant change in the post hoc test only between T0-T2 (Z=−2.45, *P*=.01). In the CF group, no statistically significant change was found across the 3 measurements (*χ*^2^_2_=1.75, *P*=.42).

#### Association Between Anxiety (STAI) to Balance Function (iTUG) and Dizziness Handicap (DHI)

There was no statistically significant correlation between STAI state and iTUG duration at any time point. However, we observed a statistically significant moderate-strong positive correlation between STAI state and DHI total in all 3 measurement sessions (Table 2). Table 2 shows the Spearman correlation between the STAI state and iTUG duration, and between the STAI state and DHI total.

**Table 2.** Correlations between anxiety scores and DHI^a^ and iTUG^b^ for all participants.

	T0^c^	T1^d^	T2^e^
STAI^f^ state and iTUG duration			
*r*	0.12	−0.36	−0.16
*P* value	.59	.19	.51
STAI state and DHI total			
*r*	0.59	0.65	0.68
*P* value	.005	.002	<.001

a DHI: Dizziness Handicap Inventory.

b iTUG: Instrumented Timed Up and Go.

c T0: first evaluation when beginning the rehabilitation period.

d T1: second evaluation after 3 weeks of rehabilitation.

e T2: last evaluation after 6 weeks of rehabilitation.

f STAI: State Trait Anxiety Inventory.

## Discussion

### Principal Findings

Twenty participants completed this 6-week study, with each subject undergoing 2 phases in random order: a period of conventional vestibular rehabilitation and a period of app-assisted vestibular rehabilitation. We examined how mobile-assisted vestibular rehabilitation affects functional balance measure (iTUG), dizziness disability (DHI), and anxiety (STAI). In addition, we examined the association between the measures (STAI with DHI and iTUG). We found that more individuals (7/9, 77%) in the AF group reached an improvement of 18 points or more on the DHI scale, indicating a clinically meaningful improvement, compared to the conventional-first group (2/8, 25%). Given the heterogeneity of vestibular diagnoses represented in our sample, it is possible that different conditions may respond differently to rehabilitation. For example, individuals with Ménière disease often show fluctuating symptom patterns [38,39], whereas patients with PPPD may require longer or psychologically oriented interventions [40]. As each diagnostic subgroup in our study included only a few participants, subgroup analyses were not feasible.

The main contributions of this paper are (1) we found that using the “Vestibulon” phone app, as part of the vestibular rehabilitation process, was associated with improvements in functional clinical indices in vestibular rehabilitation (DHI and iTUG), with a trend suggesting a possible advantage of the app-assisted condition over conventional rehabilitation; (2) we found that the correlation between the DHI questionnaire and the STAI questionnaire remained relatively constant throughout the rehabilitation period and was moderate (*r*=0.64, *P*<.001), highlighting the importance of addressing both physical and psychological aspects of vestibular disorders.

### The Effect of Using the Phone App on Functional Outcomes

The finding that DHI scores improved following vestibular rehabilitation in both groups is echoed in the literature [15,23,41,42]. Some of those studies showed greater improvement in DHI than shown in the current study; this difference may be due to the diverse population participating in our study, which included a relatively high percentage of patients (45%) with bilateral vestibular dysfunction, or PPPD, who sometimes require a more extended period of rehabilitation or a combination of additional treatments [15].

Our study revealed no significant changes in STAI scores in either group or in both groups together. This aligns with a report by Johansson et al [43], who found no effect on STAI score after vestibular rehabilitation based on home exercises combined with cognitive behavioral therapy, in a diverse cohort of vestibular patients. However, Meli et al [44] found an improvement in STAI score that was preserved after 2 months of follow-up; this was after a daily vestibular rehabilitation program supervised by a therapist, coupled with home practice, in diverse chronic vestibular patients. Our results may offer a potential explanation for these conflicting findings. We noted a distinction between participants who achieved the DHI MCID and those who did not. Specifically, those who met the MCID threshold demonstrated improvements in STAI scores, whereas those below the threshold showed deterioration (Figure 4). Although a higher proportion of participants reached the MCID during the app-assisted phase, this difference was not statistically significant (*P*=.057). Therefore, this pattern should be interpreted cautiously and viewed as preliminary, given the small sample size. Similarly, this observation is further supported by the study by Johansson et al [43], which reported no change in STAI scores and a relatively modest average change in the DHI of less than 8 points (whereas the MCID is 18). Combined, these findings suggest that vestibular rehabilitation’s impact on anxiety may be closely linked to the degree of improvement in dizziness-related symptoms, highlighting the complex interplay between physical symptoms and psychological well-being in vestibular disorders.

The results of our study suggest potential advantages of the app-assisted vestibular rehabilitation over conventional methods, although the small sample size limited our ability to demonstrate statistically significant differences. Notably, the AF group showed promising trends in several key areas. First, for DHI MCID attainment, 3 times as many participants in the AF group (7/9, 77%) met or surpassed the DHI MCID threshold compared to the CF group (2/8, 25%). Although these differences in MCID attainment did not reach statistical significance when directly comparing the 2 conditions (*P*=.057), the pattern may still indicate a potential benefit of the app-assisted approach and warrants further investigation in a larger sample. Second, for iTUG performance, only the AF group demonstrated a statistically significant improvement in the iTUG test (Figure 5), indicating a trend toward enhanced functional mobility. Third, for DHI improvement, the AF group achieved a median change of −34 (IQR 6‐35) points on the DHI, compared to −18 (IQR 10‐19) points in the CF group, potentially suggesting a more substantial reduction in dizziness-related handicap.

Furthermore, twice as many participants met or surpassed the MCID threshold of DHI while using the app (6/17, 35.2%) as participants during conventional treatment (3/17, 17.6%). While this difference did not reach statistical significance, the directionality of the findings suggests a potential advantage of the app-assisted condition. These findings, which suggest a positive trend in favor of rehabilitation with the app, need further investigation. A larger-scale study with an expanded sample size and additional outcome measures is necessary to validate the observed trends and conclusively establish whether indeed the app-assisted vestibular rehabilitation is advantageous over conventional rehabilitation.

### The "Vestibulon" App Compared to Other Apps

Several applications have been developed to enhance vestibular rehabilitation, each with unique features and limitations.

For example, D’Silva et al [26] and Meldrum et al [45] developed tablet-based apps integrated with an inertial measurement unit (IMU) sensor placed on the body. D’Silva et al [26] demonstrated high usability and improved accuracy in exercise execution compared to conventional exercises among individuals with vestibular disorders. However, their findings were based on a single session rather than a full vestibular rehabilitation program, and clinical changes were not assessed. In contrast, Meldrum at el [45] evaluated their app over an average of 8 weeks of vestibular rehabilitation and reported high usability, strong adherence, and reductions in dizziness, nausea, and anxiety. Nevertheless, they did not compare their treatment to conventional rehabilitation methods, and symptom improvement was assessed by subjective ratings rather than validated clinical tests or questionnaires typically used in vestibular rehabilitation. Hall et al [46], on the other hand, developed a vestibular rehabilitation training system incorporating a computer application and an external sensor, based on feedback from clinicians and patients. In a pilot study with individuals with vestibular hypofunction, their system demonstrated feasibility and effectiveness over a 4-week rehabilitation period. Unlike the systems mentioned above, the solution by Hall et al [46] also included functional outcomes, which provide more robust evidence of clinical effectiveness. However, similar to the systems in studies by D’Silva et al [26] and Meldrum et al [45], the system requires external hardware, such as a sensor and a computer or tablet, limiting its portability and accessibility.

A main strength of these systems is the ability to provide accurate real-time feedback through the sensors, increasing exercise precision. However, the reliance on multiple components, such as sensors, laptops, or tablets, constitutes a barrier to portability and accessibility.

Hovareshti et al [28] developed a tablet application that detects eye movement without using IMUs, using the tablet’s camera itself, and compared its capabilities with IMUs, which showed good accuracy in head angle error and mean interpeak time errors. They presented a case series examining the impact of the practice with the app on eye-gaze accuracy (the ability to maintain one’s gaze focused on a target while the head moves) in 3 patients and 1 healthy individual. They found poor eye-gaze accuracy in a participant exposed to directed energy [29]. Their significant advantage is their simplicity of use and portability, which means they do not need external sensors while providing accurate feedback based on movement detection. On the other hand, they have not yet demonstrated the app’s efficacy within the long vestibular rehabilitation process or compared it with conventional rehabilitation.

Our smartphone app was developed through a participatory-design process involving focus groups with clinicians and patients [25]. They emphasized the need for ease of use, clear instructions, feedback, cost-effectiveness, and the portability of mobile technology such as phone apps [25]. These insights guided the development of our app. We then tested the app throughout 6 weeks of vestibular rehabilitation with patients, using accepted clinical questions and functional tests.

### Association Between Dizziness Disability and Anxiety

Our study revealed a moderately positive correlation between the DHI and the STAI state scores across all 3 measurement points (*r*=0.59, *r*=0.65, and *r*=0.68 for T0, T1, and T2, respectively). These findings align with previous research on the association between DHI and STAI state. For instance, Hong et al [47] found that DHI was significantly correlated with the STAI questionnaire in a multicenter study on 407 individuals with various vestibular disorders, which examined the psychological effects of the vestibular condition. In addition, Kim et al [48] examined 544 individuals with various disorders and examined the effects of anxiety and depression on the intensity of dizziness; they found that compared to individuals with low STAI scores, individuals with high STAI scores also demonstrated higher DHI scores. Both studies examined the association between STAI and DHI at a single time point, regardless of the effect of vestibular rehabilitation. Our study extends these findings by demonstrating the correlation between DHI and STAI state at 3 points throughout the process of vestibular rehabilitation: before treatment initiation, at the midpoint, and at the conclusion of the 6-week treatment. Additionally, we observed that participants who exceeded the MCID threshold on the DHI also showed improvement on the STAI state, in contrast with those who did not reach the MCID threshold and exhibited an increase in their STAI state scores (Figure 4). These findings suggest that one possible contributor to the positive trend observed with the app-assisted condition may relate to psychological elements, such as participants’ perception of support or feedback, which have been highlighted as important by patients and clinicians [24]. However, because these factors were not directly measured in the present study, this explanation should be considered a hypothesis for future investigation. The results underscore the relationship between dizziness symptoms, functional limitations, and anxiety. The incorporation of these psychological components appears to address not only the physical aspects of vestibular rehabilitation but also the psychological factors that can impact recovery and adherence to treatment.

Interestingly, we found no correlation between STAI trait and DHI. Schuhbeck et al [49] did find an association between high STAI trait and DHI scores in the acute phase after vestibular stroke and high DHI scores at long-term follow-up. The discrepancy in the findings may be attributed to differences in study populations, as we excluded individuals with vestibular strokes.

We did not observe a statistically significant correlation between the iTUG test and the DHI scores at any measurement point (Table 2). Previous studies have reported conflicting results on the correlation between TUG and DHI, possibly due to variations in study populations and test instructions (eg, walking as quickly as possible vs at a comfortable pace). Further research is needed to clarify the relationship between DHI and iTUG, with careful consideration of population characteristics and standardized instructions.

### Limitations

This is a pilot study with a small sample size, which limits the external validity and generalizability of the findings. This study included participants with a diverse range of vestibular disorders and a broad adult age range, which offers the advantage of reflecting the clinical heterogeneity seen in practice. However, this diagnostic and age diversity may also influence the results, as certain vestibular conditions or age groups could have a disproportionate impact on specific outcomes, particularly in a small pilot sample.

Furthermore, crossover study designs present inherent challenges, as it can be difficult to ascertain which phase of the intervention contributed to the observed changes. In addition, the lack of a washout period may have introduced carryover effects between phases, and this should be considered when interpreting the comparative findings. The absence of blinding for participants, therapists, and assessors represents an additional limitation. This may have contributed to performance or detection bias, especially for subjective outcomes (DHI and STAI) and for functional assessments such as the iTUG. Although standardized assessment procedures were used, the influence of expectancy or assessor awareness cannot be fully excluded. To address these limitations, future research should implement a randomized controlled trial with a larger sample size to enhance the robustness and applicability of the results.

### Conclusions

Our findings contribute to the understanding of the interplay between dizziness, anxiety, and functional mobility in vestibular rehabilitation. The consistent correlation between DHI and STAI state scores throughout the rehabilitation process underscores the importance of addressing both physical and psychological aspects in vestibular disorders.

The “Vestibulon” smartphone app, when integrated into vestibular rehabilitation, showed improvements across several outcomes, with patterns suggesting a possible trend favoring the app-assisted phase. Taken together with the significant within-participant improvement in iTUG performance, the observation that more participants exceeded the DHI MCID threshold during the app-assisted phase suggests that integrating the “Vestibulon” app into vestibular rehabilitation may offer clinical benefits. However, these trends did not reach statistical significance and should therefore be interpreted as preliminary. These encouraging preliminary findings support the need for larger, adequately powered trials to determine the extent of the app’s contribution to rehabilitation outcomes.

## Supplementary material

10.2196/84207 Checklist 1 CONSORT checklist.

## Abbreviations

AF: app first
CF: conventional first
CONSORT: Consolidated Standards of Reporting Trials
DHI: Dizziness Handicap Inventory
IMU: inertial measurement unit
iTUG: Instrumented Timed Up and Go
MCID: minimal clinically important difference
PPPD: persistent postural perceptual dizziness
STAI: State-Trait Anxiety Inventory
