# Supplementary material for: Smartphone-Supported Vestibular Rehabilitation in Individuals With Vestibular Dysfunction: Pilot Randomized Crossover Trial Assessing Functional Clinical Outcomes and Anxiety
Source: JMIR Rehabil Assist Technol. 2026 Mar 24;13:e84207. doi: 10.2196/84207 (PMC13022543; doi:10.2196/84207)
Supplement: Checklist 1 [file rehab-v13-e84207-s001.pdf]

***CONSORT Checklist – Randomized Crossover Trial***

| SECTION/TOPIC | ITEM NO | DESCRIPTION | LOCATION IN MANUSCRIPT |
|---|---|---|---|
| **TITLE** | 1a | Identification as randomized crossover trial | Title page (p.1) |
| **ABSTRACT** | 1b | Structured, includes crossover info | Abstract (p.2) |
| **INTRODUCTION** | 2a | Background/rationale | Introduction (pp.3–5) |
| **INTRODUCTION** | 2b | Objectives/hypotheses | End of Introduction (p.6) |
| **TRIAL DESIGN** | 3a | Rationale for crossover, number/duration of periods, washout | Methods – Design (p.7) |
| **TRIAL DESIGN** | 3b | Important protocol changes | Methods – Outcomes (p.12) |
| **PARTICIPANTS** | 4a | Eligibility criteria | Methods – Inclusion/Exclusion (pp.8–9) |
| **PARTICIPANTS** | 4b | Settings and locations | Methods – Participants (p.9) |
| **INTERVENTIONS** | 5 | Details of interventions | Methods – Interventions (pp.10–12) |
| **OUTCOMES** | 6a | Prespecified primary/secondary outcomes | Methods – Outcome Measures (pp.10–12) |
| **OUTCOMES** | 6b | Changes after trial start | Methods – Outcomes (p.12) |
| **SAMPLE SIZE** | 7a | Determination | Methods – Design (p.8) |
| **SAMPLE SIZE** | 7b | Interim analyses/stopping | Not applicable (not performed) |
| **RANDOMISATION** | 8a | Sequence generation | Methods – Design (p.7) |
| **RANDOMISATION** | 8b | Blocking/stratification | Methods – Design (p.7) |
| **RANDOMISATION** | 9 | Allocation concealment | Methods – Design (p.7) |
| **IMPLEMENTATION** | 10 | Who generated/applied assignments | Methods – Design (p.7) |
| **BLINDING** | 11a | Who was blinded | Methods – Design (p.7) |
| **BLINDING** | 11b | Similarity of interventions | Methods – Interventions (p.12) |
| **STATISTICAL METHODS** | 12a | Analysis methods | Methods – Statistical Analysis (pp.14–15) |
| **STATISTICAL METHODS** | 12b | Additional analyses | Methods – Statistical Analysis (p.15) |

| | | | |
|---|---|---|---|
| **RESULTS** | 13a | Participant flow | Results + Figure 1 (p.13) |
| **RESULTS** | 13b | Numbers per sequence & period | Results + Figure 1 (p.13) |
| **RECRUITMENT** | 14a | Dates of recruitment/follow-up | Methods – Participants (pp.7–8) |
| **RECRUITMENT** | 14b | Why trial ended | Methods – Design (p.8) |
| **BASELINE DATA** | 15 | Demographic/clinical characteristics | Results – Table 1 (p.17) |
| **NUMBERS ANALYZED** | 16 | For each group/sequence | Results (pp.16–18) |
| **OUTCOMES & ESTIMATION** | 17a | Effect size/precision | Results (pp.17–22) |
| **OUTCOMES & ESTIMATION** | 17b | Binary outcomes | Results – MCID (p.19) |
| **ANCILLARY ANALYSES** | 18 | Subgroup analyses | Results – Table 2 (p.21) |
| **HARMS** | 19 | Adverse events | Results (p.16) + Abstract (p.2) |
| **DISCUSSION** | 20 | Limitations | Discussion – Limitations (p.27) |
| **DISCUSSION** | 21 | Generalisability | Discussion – Limitations (p.27) |
| **DISCUSSION** | 22 | Interpretation | Discussion/Conclusions (pp.23–28) |
| **OTHER INFORMATION** | 23 | Registration | Abstract + Methods (p.8) |
| **OTHER INFORMATION** | 24 | Protocol availability | Abstract + Methods (p.8) |
| **OTHER INFORMATION** | 25 | Funding | Declarations (p.30) |
